# Beneficial Effects of cART Initiated during Primary and Chronic HIV-1 Infection on Immunoglobulin-Expression of Memory B-Cell Subsets

**DOI:** 10.1371/journal.pone.0140435

**Published:** 2015-10-16

**Authors:** Manuela Pogliaghi, Marco Ripa, Simone Pensieroso, Monica Tolazzi, Stefania Chiappetta, Silvia Nozza, Adriano Lazzarin, Giuseppe Tambussi, Gabriella Scarlatti

**Affiliations:** 1 Università Vita-Salute San Raffaele, Milan, Italy; 2 Department of Infectious and Tropical Diseases, IRCCS Ospedale San Raffaele, Milan, Italy; 3 Viral Evolution and Transmission Unit, Division of Immunology, Transplantation and Infectious Diseases, IRCCS Ospedale San Raffaele, Milan, Italy; University of Ottawa, CANADA

## Abstract

**Introduction:**

During HIV-1 infection the B-cell compartment undergoes profound changes towards terminal differentiation, which are only partially restored by antiretroviral therapy (cART).

**Materials and Methods:**

To investigate the impact of infection as early as during primary HIV-1 infection (PHI) we assessed distribution of B-cell subsets in 19 PHI and 25 chronic HIV-1-infected (CHI) individuals before and during 48 weeks of cART as compared to healthy controls (n = 23). We also analysed Immunoglobulin-expression of memory B-cell subsets to identify alterations in Immunoglobulin-maturation.

**Results:**

Determination of B-cell subsets at baseline showed that total and Naive B-cells were decreased whereas Activated Memory (AM), Tissue-like Memory (TLM) B-cells and Plasma cells were increased in both PHI and CHI patients. After 4 weeks of cART total B-cells increased, while AM, TLM B-cells and Plasma cells decreased, although without reaching normal levels in either group of individuals. This trend was maintained until week 48, though only total B-cells normalized in both PHI and CHI. Resting Memory (RM) B-cells were preserved since baseline. This subset remained stable in CHI, while was expanded by an early initiation of cART during PHI. Untreated CHI patients showed IgM-overexpression at the expenses of switched (IgM-IgD-) phenotypes of the memory subsets. Interestingly, in PHI patients a significant alteration of Immunoglobulin-expression was evident at BL in TLM cells, and after 4 weeks, despite treatment, in AM and RM subsets. After 48 weeks of therapy, Immunoglobulin-expression of AM and RM almost normalized, but remained perturbed in TLM cells in both groups.

**Conclusions:**

In conclusion, aberrant activated and exhausted B-cell phenotypes rose already during PHI, while most of the alterations in Ig-expression seen in CHI appeared later, despite 4 weeks of effective cART. After 48 weeks of cART B-cell subsets distribution improved although without full normalization, while Immunoglobulin-expression normalized among AM and RM, remaining perturbed in TLM B-cells of PHI and CHI.

## Introduction

HIV-1 infection impairs the B-cell compartment by affecting the distribution and functionality of B-cell subsets [1–8]. Major perturbations occurring during untreated HIV-1 infection are hypergammaglobulinemia, B-cell exhaustion, impaired antigen response and alteration in the distribution of B-cell subsets [8–14]. Specifically, it is described that HIV-1 infected individuals have an increased frequency of aberrant memory B-cell phenotypes, such as Tissue-like Memory (TLM) or Activated Memory (AM) cells. Conversely, Resting Memory (RM) cells, which are responsible for an efficient secondary immune response, are depleted during the chronic stage of infection [7]. Several reports showed that these alterations are established during the early phases of the natural history of HIV-1 disease [15–18], however it has not yet been investigated whether or not these changes occur during primary HIV-1 infection.

We, as others, have shown that the timing of combined antiretroviral therapy (cART) initiation affects the recovery of B-cell compartment. cART can restore most of the B-cell subsets when given in the early phases of the disease [16–18]. Nevertheless, a complete normalization of B-cell compartment is seldom reached in successfully treated chronically infected individuals or in spontaneous viral controllers.

In physiological conditions B-cell subsets that did not encounter the antigen (i.e. Transitional and Naive cells) usually express immunoglobulin (Ig) D and IgM, while antigen-experienced B-cells (Memory and Terminally Differentiated cells) undergo somatic mutations, class switch and express one isotype only [19]. It is known that broadly cross-neutralizing antibodies, which are the result of an unusual high number of somatic hypermutations, appear in a limited percentage of HIV-1 infected individuals after several years from infection [20]. HIV-1 may perturb B-cell already during the primary phase of infection and in turn, affect maturation and Ig class switch. However, treatment during PHI seems to reduce the development of neutralizing antibodies [21].

Here we conducted a thorough analysis of B-cell subsets among HIV-1-infected patients at different timing of their natural history: particularly, in PHI and in chronic HIV-1 infection (CHI) before and after cART. First, we defined the alterations of B-cell compartment as early as in PHI. Second, to assess whether the natural history of HIV-1 infection further affected B-cells subsets we compared PHI to cART-naïve CHI patients. Moreover, we determined the impact of cART on the analyzed B-cell subsets when initiated during PHI or at a later time-point in CHI. Finally, we investigated whether HIV-1 infection could perturb Ig-maturation among memory B subsets. To clarify this issue, we described Ig-expression on memory B-cell subsets both before and after cART initiation in PHI and CHI.

## Materials and Methods

### Study groups and samples

We included 19 patients diagnosed with PHI at Fiebig stage III to V, 25 cART-naïve patients with CHI diagnosed at least 0.8 years (median 2.9, IQR 0.8–5.3 years) before enrollment, and 23 healthy controls (CS), defined as HIV-1-negative volunteers reporting no morbidities (age 23 to 67 years) (Table 1). HIV-1-infected patients were enrolled in two prospective randomised, open-label, proof-of-concept clinical trials (MAIN: http://apps.who.int/trialsearch/Trial2.aspx?TrialID=EUCTR2008-007004-29-IT; VEMAN: http://apps.who.int/trialsearch/Trial2.aspx?TrialID=EUCTR2008-006287-11-IT). The MAIN study enrolled patients with PHI, who were randomized to receive a PI-based triple-regimen with or without maraviroc (12 and 7 patients, respectively). The VEMAN study enrolled CHI cART-naïve patients randomly assigned to receive once-daily maraviroc plus lopinavir/ritonavir or tenofovir/emtricitabine plus lopinavir/ritonavir regimen (12 and 13 patients, respectively).

**Table 1:** Baseline characteristics of study participants. PHI are individuals recruited at Fiebig stage III to V of HIV-1 infection before donating baseline samples. CHI are chronically HIV-1 infected individuals having been infected for at least 0.8 years (median 2.9, IQR 0.8–5.3 years,) and not receiving cART at recruitment. MSM means Men having sex with men; IVDU means intravenous drug user; WBC means White Blood Cells; AST means aspartate aminotransferase; ALT means alanine aminotransferase. Statistical analysis was performed using (a) Mann-Whitney test and (b) Chi-square test. P values <0.05 were considered significant differences.

	Value for group, Median (IQR) or No. (%)		P-value
	PHI	CHI	
**No. of participants**	19	25	
**Age, years**	39 (33–41)	40 (35–47)	0.235 ^(a)^
**Gender**			0.842 ^(b)^
**Female**	1 (5.3)	1 (4)	
**Male**	18 (94.7)	24 (96)	
**Risk factor**			0.976 ^(b)^
**MSM**	13 (68.4)	17 (68.0)	
**Heterosexual**	6 (31.6)	8 (32)	
**Fiebig stage**			
**III**	7 (37)	Not applicable	
**IV**	1 (5)	Not applicable	
**V**	11 (58)	Not applicable	
**HIV-RNA, log10 copies/mL**	5.8 (5.2–6.4)	4.2 (3.7–4.8)	**<0.001** ^**(a)**^
**WBC, cells/uL**	6600 (5100–8300)	5000 (4350–6350)	**0.017** ^**(a)**^
**Neutrophils,cells/uL**	3000 (2600–3300)	2700 (2100–3250)	0.307 ^(a)^
**CD4+ T cells, cells/uL**	381 (266–470)	282 (249–333)	**0.049** ^**(a)**^
**CD8+ T cells, cells/uL**	1573 (884–2697)	1028 (717–1315)	**0.016** ^**(a)**^
**CD4/CD8, ratio**	0.17 (0.15–0.42)	0.32 (0.20–0.40)	0.052 ^(a)^
**Hemoglobin, g/dL**	13.8 (13.3-14-8)	14.4 (13.8–15.1)	0.058 ^**(a)**^
**Platelets, cells/uL**	181000 (160000–247000)	200000 (155000–240000)	0.307 ^(a)^
**Creatinine, mg/dL**	0.86 (0.70–0.95)	0.83 (0.79–0.97)	0.767^(a)^
**AST**	28 (20–65)	23 (30–31)	0.217^(a)^
**ALT**	46 (31–108)	29 (22–53)	**0.022** ^**(a)**^

The Institutional Review Board approved both studies (MAIN: 67/DG-20/2/2009, VEMAN: 417/DG-8/5/2009) as well as the immunological sub-studies described herein. All patients provided written informed consent approved by the Ethics Board according to national legislation.

Peripheral blood mononuclear cells (PBMCs) were isolated by Ficoll Gradient separation (Lymphoprep Nycomed, Axis-Shield) from venous blood collected from study individuals at baseline (BL), week (W) 4 and W48 of cART, and from healthy controls, were frozen and stored in liquid nitrogen until experiments were performed. Samples of each individual were tested simultaneously.

HIV1-RNA plasma viral load was quantified using the Abbott m2000 RealTime System, with a detection limit of 40 copies/mL.

### B-cell subsets analysis

Multi-color flow cytometry was performed on PBMC by acquiring 106 formaldehyde-fixed cells at the Gallios (Beckman Coulter) flow cytometer, and data analysed with FlowJo 8.8.7 (Treestar Inc.). The following mouse antihuman fluorochrome-conjugated monoclonal antibodies were used: CD19-PER-CP-Cy5.5 (SJ25-C1), CD10-PE-Cy7 (Hl10a), CD38-APC (HIT2), CD21-PE (B-ly4), CD27-V450 (M-T271), IgD-APC-H7 (IA6-2) and IgM-FITC (G20-127) (BD Biosciences). Dead cells were excluded using the Live/Death Vivid detection kit labeled with a near-infrared dye (Invitrogen, Carlsbad, California, USA). Gating strategy was performed as follows: only singlets were acquired and live B cells were gated on Vivid-CD19+ lymphocytes. Only samples with viability above 85% were subjected to the B-cell analysis. Transitional (“TR”) B cells were identified as CD19+CD27-CD10+. Thereafter, the CD10- cell population was gated on CD27++CD38high+ to identify Plasma-cells. Whereas on the CD27+CD38high- gate the following populations were identified: naive (“NV” CD27-CD21high+), resting memory (“RM” CD27+CD21high+), activated memory (“AM” CD27+CD21-) and tissue-like memory (“TLM” CD27-CD21-). RM, AM and TLM were each further characterized on the basis of Ig-expression into switched (“SW”, IgM-IgD-), Marginal Zone-like (“MZL”, IgM+IgD+), IgM-expressing (“IgM+”, IgM+IgD-) and IgD-expressing (“IgD+”, IgM-IgD+).

### Statistical analysis

Results were described as median (interquartile range, IQR) or frequency (%), as appropriate. Intention-to-treat principle was used for analysis. Mann-Whitney and Kruskal-Wallis tests were used to compare values of continuous distributions. Significant changes of continuous variables were evaluated by the Two-sample paired sign test. Linear correlations were assessed by the Spearman rank correlation coefficient. A P-value<0.05 was considered as significant. Statistical analysis was performed by SPSS (release 20, IBM).

## Results

### Viro-immunological follow-up of study groups

Two groups of HIV-1-infected individuals, PHI and CHI, including 19 and 25 individuals respectively, were studied besides the control group (CS, n = 23). Data set is available as (S1 File). PHI and CHI individuals were, as expected, at BL intrinsically different concerning some immune-virological parameters, but were comparable for characteristics unrelated to timing of HIV infection (Table 1) and did not present any other comorbidities or hepatic virus co-infections.

**Fig 1:**
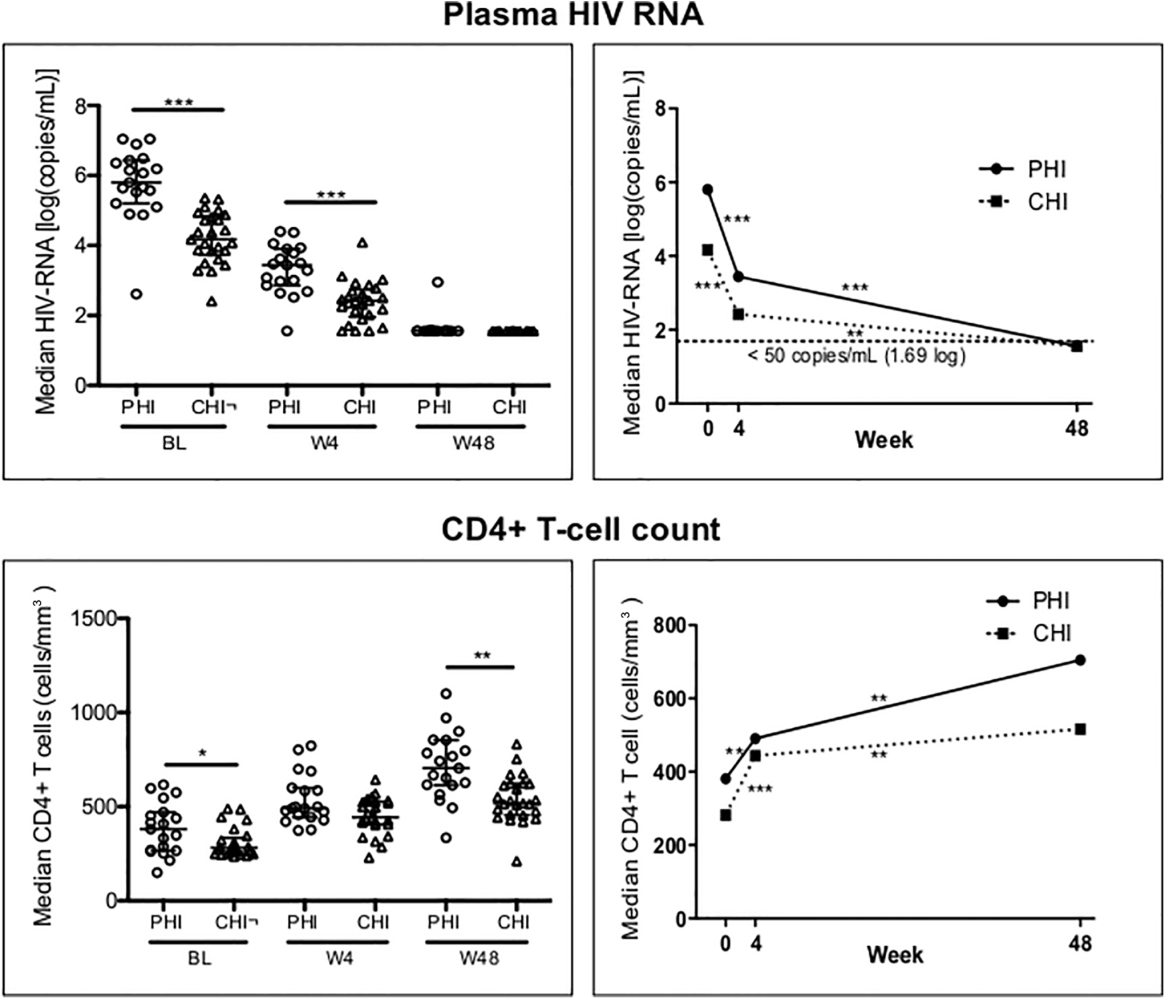
Viro-immunological follow-up of study groups. CD4+ T-cell count (cells/mm^3^) and plasma viremia (log copies/ml) for PHI and CHI patients are here represented. Left panels show medians with IQR of each parameter; right panels show median values of each time-point to highlight the trend over time. P values are * = 0.01–0.05, ** = 0.001–0.01, *** = <0.001, obtained with Mann-Whitney test for left panels and with Two-sample paired sign test for right panels.

As expected, PHI had significantly higher plasma HIV-1-RNA load and CD4+ T-cell count at BL in comparison to CHI (Table 1 and Fig 1). Notably, alanine aminotransferase (ALT) were higher in PHI patients, probably due to the hepatic involvement during PHI (Table 1). cART was started for both PHI and CHI with regimens specific to each study protocol as described in Materials and Methods section. HIV-1-RNA load decreased by 4 weeks of treatment in both groups, but still remained higher in PHI compared to CHI (Fig 1). Virological suppression, defined as plasma HIV-1-RNA below 50 copies/ml, was obtained at 48 weeks in 18 out of 19 PHI and in all CHI. After cART initiation CD4+ T-cell count increased in the two groups through-out the whole follow-up reaching similar levels at 4 weeks, whereas at 48 weeks PHI had higher CD4+ T-cell counts than CHI (Fig 1). No differences in terms of CD4+ T-cell gain or in decrease of HIV-1-RNA load were observed between the different cART regimens applied (data not shown).

### PHI drives B-cell subsets towards terminal differentiation

**Fig 2:**
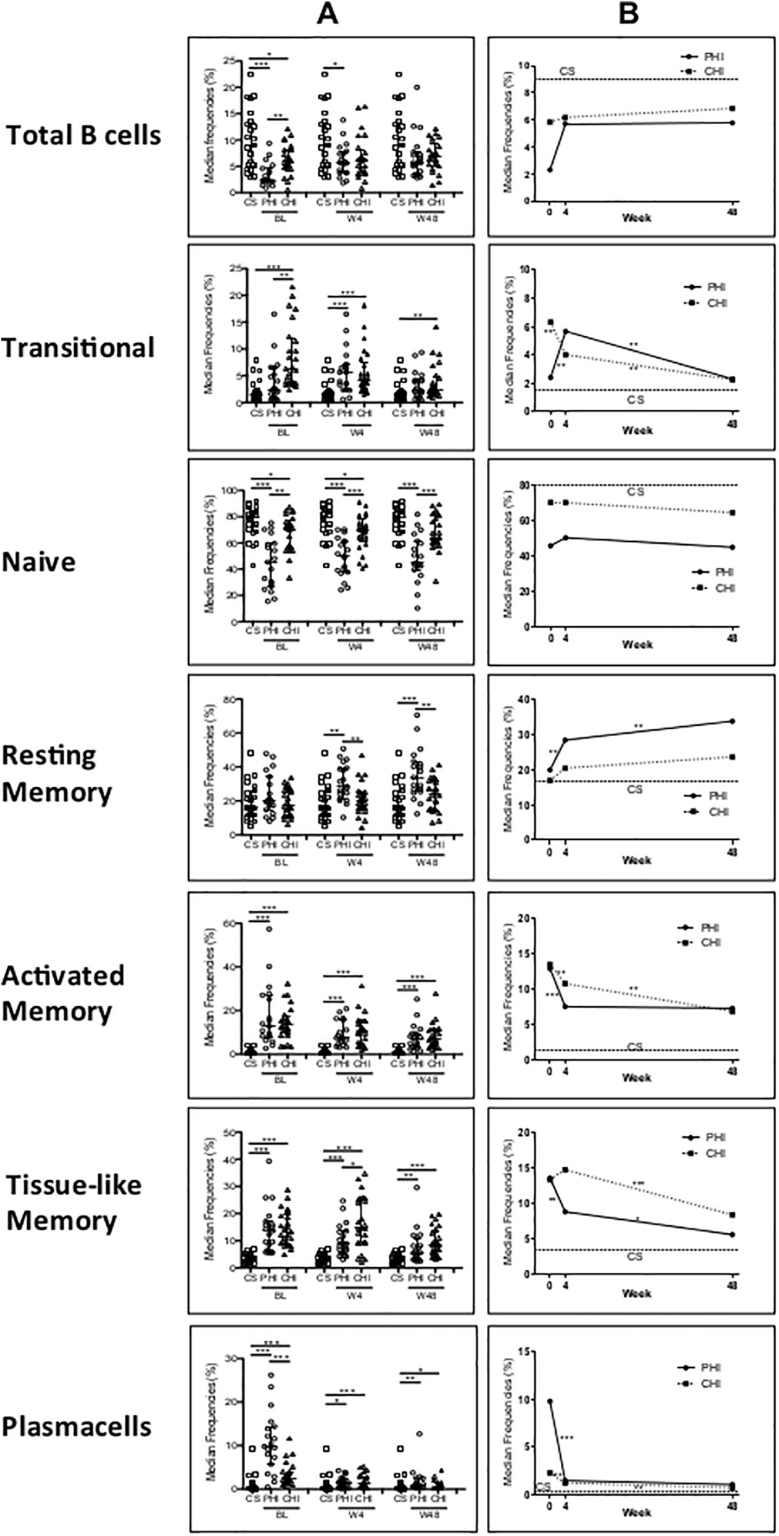
B-cell subsets distribution and trend of study groups. Indicated are frequencies of B-cells subsets in PHI and CHI at baseline and after 4 and 48 weeks of cART, as well as values of healthy controls (CS). Column A shows medians with IQR of each parameter; Column B shows median values of each time-point to highlight the trend over time. Dotted horizontal line represents the mean of the CS individuals for a given cell population. P values are * = 0.01–0.05, ** = 0.001–0.01, *** = <0.001, obtained with Mann-Whitney test for results of column A and with Two-sample paired sign test for results of column B.

Given previous reports that HIV-1 infection is associated with increased terminal differentiation of B-cells [3, 22, 23], we wished to determine the impact on B-cell subsets of PHI in comparison to CHI as well as to healthy controls. Already at BL the frequencies of total B-cells of PHI individuals were significantly lower compared to those of CHI and healthy controls (Fig 2, panel A). Moreover, some of the major B-cell subsets of both PHI and CHI were significantly different compared to those of healthy controls. Specifically, frequency of the immature TR B-cells was preserved in PHI, but was increased in CHI compared to healthy controls. Whereas NV B-cells, the most represented compartment among healthy controls, were decreased in PHI compared to CHI and healthy controls. RM B-cells were preserved and had comparable frequencies in PHI and CHI. Interestingly, AM and TLM B-cells were increased in PHI to levels to those of CHI. Finally, PHI had a significantly expanded pool of Plasma cells compared to CHI, while, as expected, in healthy controls this subset constituted a negligible portion. Thus, a differentiation toward exhausted B-cells subsets and Plasma cells is already evident during PHI.

### Recovery of B-cell compartment during cART is not complete in PHI and CHI

We therefore wanted to test if cART could restore B-cell compartment in acute infection as compared to CHI. cART had a relevant impact on B-cells already after 4 weeks (Fig 2, panel B). In PHI total B-cells did not significantly change from BL, remaining lower than in healthy controls. NV B-cells did not change over time, and persisted depleted in both PHI and CHI, with the lowest frequency among PHI. Interestingly, TR B-cells, which were not affected at BL in PHI, showed a steep rise, while they declined in CHI (Fig 2, panel B). Thus, at week 4, TR B-cells reached comparable frequencies among the two groups of HIV-1-infected patients, still remaining higher than in healthy controls. Surprisingly, RM B-cells, which were preserved at BL in PHI, significantly increased to reach higher frequencies than in CHI and healthy controls (Fig 2, panel A). Among the exhausted B-cell subsets, AM significantly decreased in both PHI and CHI, while TLM decreased only in patients with acute infection. Plasma cells showed a significant decrease in both PHI and CHI (Fig 2, panel B). Nevertheless, frequencies of these three latter B-cell subsets were still higher in PHI and CHI compared to healthy controls (Fig 2, panel A).

At week 48 of cART, total B-cells became comparable in both groups to healthy controls. The B-cell subsets underwent further modifications. While NV B-cells in CHI became comparable to healthy controls, this subset remained lower in PHI than in both healthy controls and CHI (Fig 2, panel A). In PHI TR B-cells, after an initial rise at week 4, decreased, as observed in CHI. Surprisingly, RM further increased in PHI, but not in CHI (Fig 2, panel B). The exhausted AM pool continued to decrease significantly only in CHI, while TLM decreased in both PHI and CHI (Fig 2, panel B). In the end, despite a substantial decrease after 48 weeks of cART, AM and TLM subsets failed to reach normal values (Fig 2, panel A). Finally, Plasma cell frequencies did not change in PHI, but further decreased since week 4 in CHI. However, Plasma cells remained slightly higher in both groups of HIV-1-infected patients compared to healthy control values (Fig 2, panel A). In conclusion, in spite of a long-term effective cART and a recovery of the total B-cell pool, TLM, AM and Plasma cells did not drop to reach normal levels neither in PHI nor in CHI individuals. In PHI the RM pool remained surprisingly expanded, possibly at the expenses of the Naïve B-cells.

### Correlations between viro-immunological parameters and B-cell subsets

As we previously published [18] a correlation between viral load or CD4+ T-cell counts and the B-cell subsets distributions, we investigated these parameters at BL and after cART initiation. Plasma viral load of PHI at BL correlated with frequencies of total B cells and Plasma cells (r = -0.499, p = 0.030, and r = 0.519, p = 0.023, respectively). Drop at week 4 and week 48 of plasma HIV-1-RNA load of PHI patients correlated with a decrease in Plasma cells (r = 0.465, p = 0.045 and r = 0.647, p = 0.003 respectively). CD4+ T-cell count at BL was inversely correlated with Plasma cells percentages in PHI (r = -0.475, p = 0.040) and with TR B-cells in CHI (r = -0.474, p = 0.017). In PHI, after 4 weeks of cART the increase of CD4+ T-cells correlated with the decrease of TLM B-cells (r = -0.485, p = 0.035). No further correlations were found in CHI patients. In conclusion, we showed that a decrease in PHI Plasma cells was directly correlated to HIV-1-RNA decline, highlighting a possible role of the virus in B-cell activation.

### Ig-expression on memory B-cell subsets

Our finding that memory and differentiated B-cell subsets were increased in PHI and CHI prompted us to investigate if the expression of immunoglobulins on these B-cell subsets were affected as well. To this end, we assessed Ig-expression on RM, TLM and AM B-cells, identifying four subpopulations: switched B-cells (IgM-IgD-), IgM-expressing (IgM+IgD-), IgD-espressing (IgM-IgD+) and MZ-like B-cells (IgM+IgD+). Switched B-cells classically represent a subset already exposed to a known antigen, while IgM+ or IgD+ show phenotypes characteristic of immature or naïve cells. We also analyzed the double IgM and IgG expressing cells, which are usually detected in the MZ of the lymph-node, and are thus called MZ-like cells.

**Fig 3:**
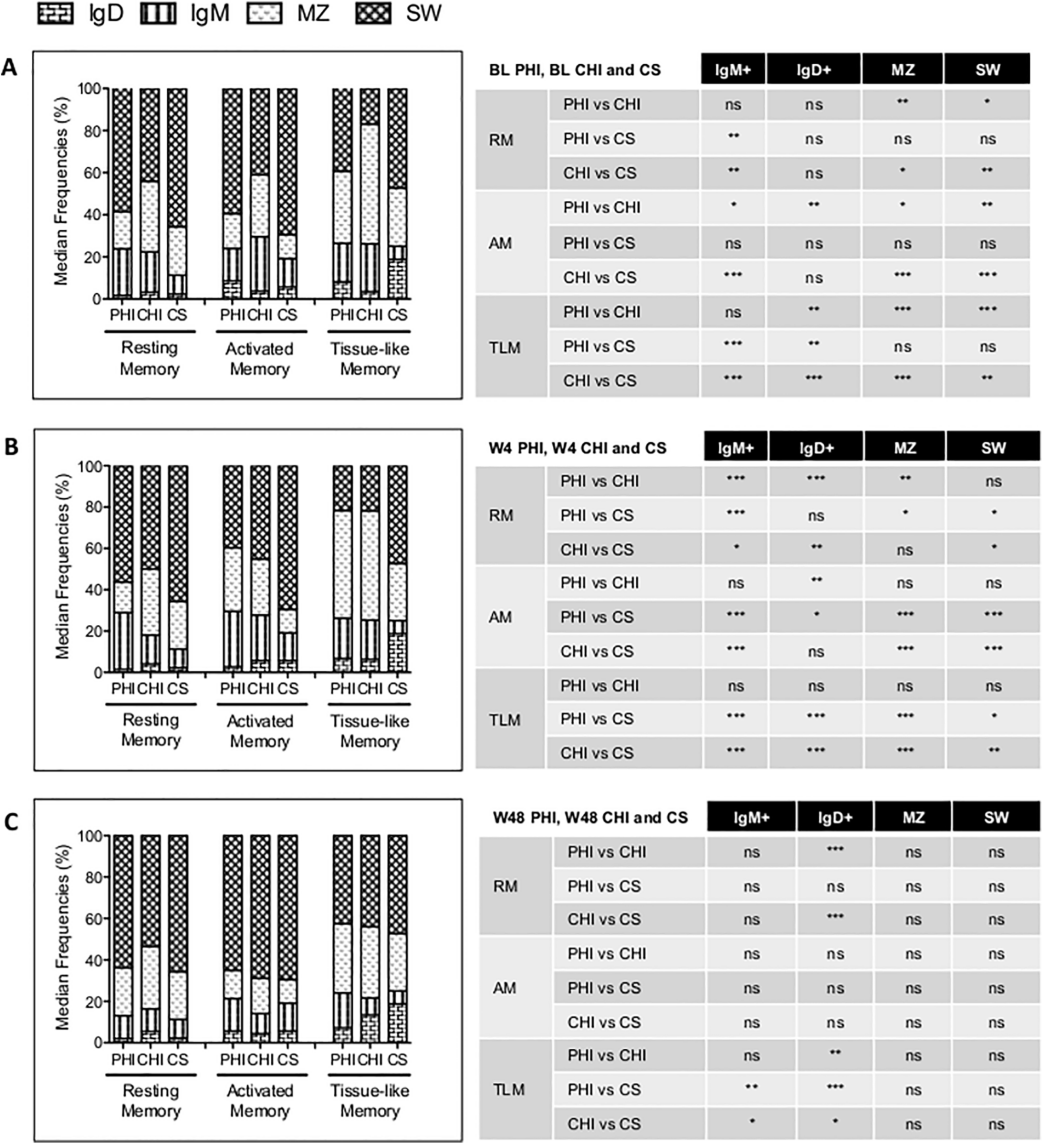
Immunoglobulin expression of B-cell subsets. Indicated are frequencies of Ig-expression on RM, AM and TLM B-cells in PHI, CHI and CS. Ig-expressing B-cells are defined as IgM-only “IgM+” (IgM+IgD-), IgD-expressing “IgD+” (IgM-IgD+), Marginal-Zone like “MZ” (IgM+IgD+) and Switched “SW” (IgM-IgD-) B-cells. The column plots to the right represent the median frequencies of Ig-expression in the B-cell subsets, the tables to the left indicate p-values of comparisons (* = 0.01–0.05, ** = 0.001–0.01, *** = <0.001, ns = not significant, Mann-Whitney test). A. Frequencies of PHI and CHI individuals at BL in comparison to CS. B. Frequencies of PHI and CHI individuals at week 4 (W4) in comparison to CS. C. Frequencies of PHI and CHI individuals at week 48 (W48) in comparison to CS.

We first determined the frequencies of these phenotypes in healthy controls (Fig 3). SW cells, as expected, were the major compartment among all Memory B-cell subsets, while MZL were the second most represented population. IgM+ were the third compartment, except for TLM, where they were the least represented subset. Finally, IgD+ compartment was the least represented among AM and RM B-cells.

We then proceeded to investigate how HIV-1 infection could affect Ig-expression (Fig 3, panel A). At BL, in PHI SW and MZL cells were not significantly affected, while individuals with CHI had decreased SW and increased MZL phenotypes in all three B-cell subsets. IgM+ cells were significantly increased in RM and TLM of PHI and in all three B-cell subsets of CHI. IgD+ cells were significantly increased to similar degree in both PHI and CHI but only in TLM cells. Thus, during PHI Ig-expression of the B-cell compartment was already affected, especially in TLM, which expressed higher levels of IgM+ and IgD+, and RM cells, which also had higher frequencies of IgD+. On the other hand, CHI had major perturbations among all memory B-cells subsets, with preservation of IgD+ among RM and AM uniquely.

### Recovery of Ig-expression on B-cell subsets during cART

cART restored to a certain degree the total B-cell pool and some of the subsets. Thus, we wished to define whether or not Ig-expression was normalized on memory B-cells subsets, RM, AM and TLM B-cells.

#### Switched cells (SW)

**Fig 4:**
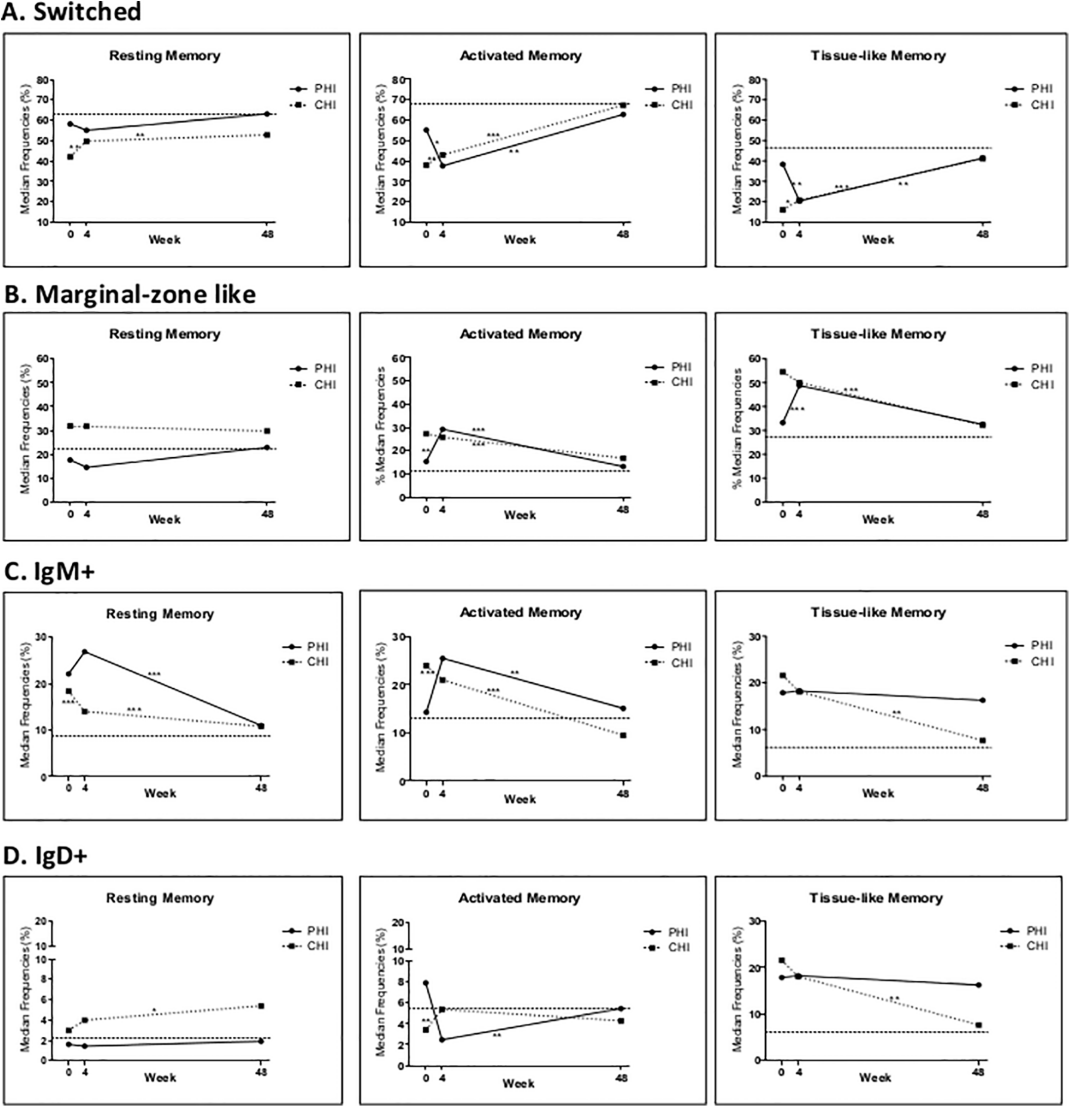
Evolution in Immunoglobulin expression of B-cell subsets in study groups vs CS. Each panel represents changes over time of different Ig-subsets in Resting Memory, Activated Memory, and Tissue-like Memory B-cell of PHI and CHI individuals: A. Switched cells; B. Marginal zone-like cells; C. IgM+ cells; D. IgD+ cells. Dotted horizontal line represents the mean of the CS individuals for a given cell population. P values are * = 0.01–0.05, ** = 0.001–0.01, *** = <0.001, obtained with Two-sample paired sign test.

After 4 weeks of cART, in PHI SW cells significantly dropped in AM and TLM B-cells and did not change in RM cells (Fig 4, panel A). Interestingly, SW cells in PHI patients reached levels comparable to those observed in CHI individuals at BL (Fig 3, panel A and B). Conversely, in CHI all SW Memory subsets significantly raised (Fig 4, panel A), although not reaching frequencies seen in healthy controls. At week 48 of cART, SW cells increased in most of memory B-cell subsets of both PHI and CHI (Fig 4, panel A), finally reaching levels comparable to healthy controls for all memory subsets (Fig 3, panel C).

#### Marginal zone-like cells (MZL)

After 4 weeks, in PHI MZL cells of AM and TLM B-cells significantly increased (Fig 4, panel B), showing values similar to those of CHI patients at BL (Fig 3, panel A and B). On the other hand, in CHI MZL cells remained stable (Fig 4, panel B). At Week 48, in both PHI and CHI, MZL cells of AM B-cells significantly decreased from Week 4 (Fig 4, panel B), while MZL cells of TLM-B cells decreased significantly only in CHI, all reaching levels comparable to those of healthy controls (Fig 3, panel C).

#### IgM-expressing cells (IgM+)

At week 4, in PHI, IgM+ cells remained stable (Fig 3, panel B and Fig 4, panel C). On the contrary, in CHI individuals IgM+ AM and RM cells started to decrease. These changes in CHI, although leading towards a normalization of Ig-expression on memory B-cell subsets, failed to recover to reach levels of healthy controls. At week 48, in PHI IgM+ AM and RM cells decreased from week 4, as in CHI. IgM+ TLM cells did not change in PHI, while they significantly decreased in CHI (Fig 4, panel C). Eventually, after prolonged cART AM and RM B-cell subsets normalized the IgM+ pool, while TLM cells remained altered in both PHI and CHI.

#### IgD-expressing cells (IgD+)

At BL IgD+ cells were significantly increased only in TLM cells to a similar degree in PHI and CHI. After 4 weeks of cART, in PHI IgD+ cells decreased in TLM (Fig 4, panel D), whereas they remained stable in RM subset. Consequently, at week 4 in PHI IgD+ AM cells became lower than in healthy controls (Fig 3, panel B). Instead, in CHI IgD+ in AM and TLM cells significantly increased (figure D). At week 48, IgD+ AM cells did not change in CHI, while they significantly increased in PHI (Fig 4, panel D), becoming comparable to those of healthy controls (Fig 3, panel C). IgD+ TLM cells did not change in PHI and increased in CHI (Fig 4, panel D). Thus, for both patients groups these cells failed to normalize (Fig 3, panel C). Interestingly, IgD+ RM cells did not change over time in PHI, while in CHI there was a significant increase from week 4 to week 48 (Fig 4, panel D). Thus, IgD+ RM B-cells in PHI individuals remained stable at comparable levels to healthy controls but in CHI individuals were higher at week 4 (Fig 3, panel C).

## Discussion

Our results show that B-cell populations are already affected during PHI. Patients with acute infection had the lowest frequencies of NV B-cells and the highest percentages of Plasma cells compared to CHI and healthy controls, probably reflecting the massive immune-activation triggered by the recent encounter with the new pathogen and its enormous levels of replication [6, 22, 24]. Interestingly, also AM and TLM B-cells, despite the short time since HIV-1 transmission, were heavily perturbed at levels comparable to those of CHI, demonstrating that so-called aberrant activated and exhausted B-cell phenotypes rise as early as during PHI.

We cannot exclude that other factors apart from the virus may contribute to the modifications of B-cells subsets and the expansion of Plasma cells during PHI. Indeed, Moir and colleagues showed that B-cells up-regulate several type I interferon-induced genes as well as genes associated with B-cell terminal differentiation [25]. Furthermore, several soluble factors induced during HIV-1 Infections, including IP-10, IL-10 and ICAM, have been associated with B-cell activation or terminal differentiation [26, 27].

RM B-cells had similar frequencies in both groups of HIV-1 infected patients compared to healthy controls. In previous studies Authors [3, 28, 29] found lower levels of resting memory in CHI compared to healthy controls and only a partial recovery of this subset under cART. Our group has already shown a preservation of RM cells among naive CHI [18]. Interestingly, in this last work the highest frequency of RM B-cells was observed among PHI after only 4 weeks of therapy and at the end of follow-up. We may envisage that early cART initiation could have played a role in preserving or even expanding the pool of this subset. The conservation of this crucial memory subset [17], which we also found to be elevated in HIV-1 elite-controllers compared to chronic naïve and experienced patients, could be beneficial in preserving an efficient humoral response against pathogens.

The early changes of B-cell subsets we observed prompted us to think that HIV-1 during the primary phase of infection may also affect B-cell maturation and Ig-class switching. For this purpose, we assessed Ig-expression in B-cell subsets representative on one side of the aberrant phenotypes, the AM and exhausted TLM cells, and on the other side of the RM cells, which classically drive the memory antibody response to antigens. This is the first report describing in detail the Ig-expression of B-cell subsets in HIV-1 infected and uninfected individuals. In uninfected individuals the switched phenotype (IgM-IgD-) was the major subset, while IgM+, IgD+ and MZL phenotypes, which characterized immature and lymph-node circulating B-cells, were expressed to a lesser extent. In PHI patients only TLM B-cells had major alterations in Ig-expression, with an overexpression of IgM+ and IgD+, i.e. phenotypes characteristic of immature or naïve cells. Conversely, in CHI cART-naive patients, we observed a change of Ig-expression in all memory B-cell subsets: specifically, IgM+ and MZL cells were overrepresented at the expense of SW B-cells in all subsets. Our data are in line with those described by Moir at al. [2] in CHI untreated viremic patients: HIV-1-specific Antibody-Secreting Cells (ASCs) were enriched in TLM B-cells in HIV-1-infected individuals. In this subset, frequencies of IgM and IgG/A ASCs were similar. Moreover, they reported that the frequencies of IgG/A ASCs were significantly higher than IgM ASCs in the non-aberrant memory B-cell population.

Interestingly, in patients with PHI we documented a difference in trend between the first 4 weeks after cART introduction and the subsequent 44 weeks. Specifically, following modifications of most B-cell subsets after the first 4 weeks, a trend towards normalization was apparent in the majority of B-cell populations. The exception, as discussed before, was the TLM B-cell subset, being altered since BL even for acute patients. In CHI patients 48 weeks of cART drove towards an almost full normalization of Ig-expression among memory B-cell subsets. In both PHI and CHI we observed major alterations of B-cell subsets distribution. Conversely, Ig-expression among memory B-cell subsets was not deeply perturbed as in CHI at the onset of HIV-1 infection. It is noteworthy that, despite an efficient cART during the first 4 weeks, persistent HIV-1 replication led to the same changes observed in untreated CHI. This finding may be explained by the inability of cART to fully suppress viral replication in such a short time, thus allowing the virus to continue the damage induced on Ig-expression, which may take longer to occur than major subsets alterations. Our observations raise the question of how early cART has to be initiated to effectively contain alterations of B-cells. Indeed, recognition of the acute infection is rare and thus, precludes a timely initiation of therapy.

After 48 weeks of effective therapy, all B-cell phenotypes had reverted their trend in PHI and CHI but most B-cells did not reach levels comparable to those of healthy controls. Indeed, PHI restored normal levels of TR and CHI those of NV B-cells, while Plasma cells, TLM and AM remained over-expressed in both groups. Finally, Ig-expression almost fully recovered after 48 weeks of cART in AM and RM subsets. Nevertheless, Ig-expression of TLM remained perturbed in both PHI and CHI when compared to healthy controls. In particular, we found a persistent over-expression of IgM+ and under-representation of IgD-expressing subsets, which were also the first ones subverted during PHI. We previously showed that B-cells are still affected after 24 weeks but are restored after 144 weeks of therapy in a multiple-failure cohort treated with a raltegravir-containing regimen [18]. Thus, further studies are needed to evaluate the effect of cART beyond 1 year of treatment.

In conclusion, we have demonstrated that B-cell alterations are established shortly after HIV-1 infection. A thorough analysis showed that Ig-expression of TLM B-cells were the first affected and remained altered even after 1 year of efficient cART. It may be of interest to investigate whether this subpopulation of TLM, which is particularly resistant to cART, could play a role in the deferred neutralizing antibody response characteristic of HIV-1 infection. Treatment during PHI caused a significant increase of CD4+ T-cell count and seemed to preserve the RM pool, fundamental for secondary humoral immune response. This gain was not observed in CHI, suggesting a potential further benefit in starting cART as soon as possible in the natural history of HIV-1. Finally, our data indicated that full restoration of B-cells could be a long-lasting process. Thus, long-term follow-up is needed to assess whether early cART initiation could bring additional advantages among B-cell compartments and if this could be translated into a clinical benefit.

## Supporting Information

S1 File Data set used for statistical analysis of our work. Column A identifies the study group (PHI patients: MAIN, CHI patients: VEMAN, HC: healthy controls). Column B identifies the code of each patient who participates to the studies. Column C identifies the Fiebig stage for PHI patients. Columns D, Z and BA identify the absolute CD4+ T cell count (expressed as cell count/mm3) at BL, W4 and W48 respectively, for HIV patients (MAIN, VEMAN). Columns F, AB and BC identify plasma viremia (expressed as HIV-RNA copies/ml) at BL, W4 and W48 respectively, for HIV patients (MAIN, VEMAN). The other columns represented frequencies of CD4+ T cell, B-cells subsets and Ig-expression of memory B-cells among all groups. Columns in yellow represent BL. Columns in blue represent W4. Columns in green represent W48.
(XLS)
